# Emerging Field-Effect Transistor Biosensors for Life Science Applications

**DOI:** 10.3390/bioengineering10070793

**Published:** 2023-07-02

**Authors:** Abbas Panahi, Ebrahim Ghafar-Zadeh

**Affiliations:** Biologically Inspired Sensors and Actuators, Lassonde School of Engineering, York University, Toronto, ON M3J 1P3, Canada; panahiyu@yorku.ca

Field-effect transistors (FETs) have gained significant interest and hold great potential as groundbreaking sensing technology in the fields of biosensing and life science research. Researchers are captivated by the remarkable features offered by FETs, such as miniaturization, parallel sensing capabilities, rapid response times, and seamless integration with electronic manufacturing processes, including complementary metal-oxide semiconductors (CMOSs) [1,2,3]. The origins of FET-based sensing techniques can be traced back to the 1970s when the ion-sensitive FET (ISFET) was developed, stemming from the pioneering work on metal-oxide-semiconductor FETs (MOSFETs) by Bergveld. This historical trajectory marks the foundation for the remarkable progress and innovation witnessed in the field of FET-based sensing [4]. Initially focusing on detecting hydrogen ions, these pioneering developments paved the way for subsequent breakthroughs and expanded applications in biomolecular sensing [4]. The capability of FETs to detect ions and explore biomolecular interactions has been a driving force behind the advancements in gene detection and cellular/biomolecular sensing. One particularly promising aspect of FET-based sensing is its potential to revolutionize single-molecule analysis in biology. Through the utilization of FETs for miniaturized and parallel sensing, researchers can obtain real-time, high-resolution data on the concentration/activity of target analytes or the presence/quantity of biomolecules. The rapid response times and seamless integration of FETs with electronic manufacturing processes further enhance their attractiveness in point-of-care devices for at-home diagnostics [5,6,7].

Moreover, there has been notable progress in utilizing FET-based sensing techniques to detect and study genes. Researchers have explored various gate-modification techniques to expand the capabilities of FETs in this domain [8]. This ability to interrogate and comprehend the intricate interactions between genes opens up new avenues in life science research, particularly for virus detection and the development of amplification-free-virus gene fingerprinting. FETs serve as a distinctive platform for investigating biomolecular interactions with remarkable sensitivity, precision, and cost-effectiveness by measuring the inherent charge of genes during hybridization and their direct interaction with the FET’s sensing gate. Harnessing the unique capabilities of Gene-FETs, researchers can gain valuable insights into ultra-small molecules that are challenging to measure and single gene mutations by utilizing size-comparable FET structures like nanowires and graphene FET sensors. This paves the way for breakthroughs in diagnostics, therapeutics, and drug development [9].

The prospects of FETs in life science research are vast and promising. The remarkable progress achieved in FET-based sensing techniques has propelled the development of diverse sensor structures and sensing materials. This multitude of sensor system combinations offers unprecedented opportunities for detecting and monitoring various target analytes in complex biological samples. Furthermore, integrating FETs with advanced data analysis techniques and connectivity with cloud-based platforms enables data-driven precision medicine and collective knowledge-sharing [10]. By exploring the historical evolution and current state of the art of FETs, this editorial paper sheds light on the immense promise of this technology in revolutionizing precision medicine and life science research. The comprehensive overview presented here serves as a testament to the transformative power of FETs and their invaluable contribution to revolutionizing precision medicine and the advancement of the life sciences.

## 1. Advantages and Applications of BioFET Sensors

Field-effect transistor (FET) biosensors offer several advantages, which have contributed to their wide range of applications. Firstly, FET biosensors enable label-free detection, eliminating the need for fluorescent tags that can introduce complexities and potential interference in other sensing mechanisms like optical methods. This attribute simplifies the sensing process, reduces costs, and enhances compatibility with various analytes. For example, George Alexandrou et al. [11] developed a microfluidics-integrated ISFET-based biosensor for the ESR1 mutations as an essential biomarker in metastatic breast cancer. Exploiting the unique advantage of a highly charge-sensitive silicon-based BioFET, they successfully distinguished wild-type (WT) from mutant (MT) DNA in ESR1 p.E380Q and p.Y537S targets in a completely label-free method. FET biosensors demonstrate high sensitivity, allowing for the detection of low analyte concentrations. This sensitivity is attributed to the inherent electrical amplification of FET devices, which can amplify the small changes in the charge distribution near the sensor surface. Jae-Hyuk Ahn et al. developed a profound theoretical understanding of dual-gate sensing on a FET sensor in carrying out pH measurements beyond the Nernstian limit with a FET sensor using both a solution and back gate [12]. Jin-Hyeok Jeon and Jin-Hyeok Jeon fabricated a high-performance dual-gate ISFET that obtained a pH sensitivity of 304.12 mV/pH, which can only be seen in FET structures that amplify bio/chemical signals in the device before sending data to the readout circuit [13]. In terms of applications, FET biosensors have found utility in diverse fields, as we outlined in a thorough review in another work [9]. In the medical diagnosis of infectious diseases, which was needed in the recent pandemic, FET biosensors show promise for point-of-care testing, early disease detection, and monitoring treatment responses. We have provided a complete review of the state of the art of FET biosensors for infectious disease detection in another work, introducing all FET structures, materials, and readout circuits [8]. The high sensitivity of BioFETs and the ability to control it through the semiconductor structure design, surface chemistry, and readout circuits are unparalleled advantages.

In light of the previous state-of-the-art advancements, we believe FET biosensors offer advantages such as label-free detection and high sensitivity; however, their application in various fields requires critical assessment. In the next section we provide examples from the literature demonstrate the potential of FET biosensors to achieve these advantages in specific applications.

## 2. Emerging Trends in BioFET Research

FET-based biosensors are witnessing exciting advancements driven by emerging trends and future directions, with tremendous potential for enhancing their performance and expanding their applications. One prominent trend is the exploration of novel materials in FET biosensors. Two-dimensional materials, such as graphene, transition-metal dichalcogenides, and black phosphorus, offer unique electrical properties and high surface-to-volume ratios [14]. These materials promise to improve the sensitivity, selectivity, and stability of FET biosensors. Researchers are actively investigating their integration into biosensor platforms and functionalization techniques that modify the sensor surface with bioactive molecules or nanoparticles, enabling enhanced specificity and signal transduction [15]. Using novel materials in FET biosensors is poised to drive significant advancements in their performance and open up new possibilities in healthcare, diagnostics, and biotechnology [14]. However, research on their scalability, material stability in complex solutions, and reproducibility are still challenges when preparing them for real-world applications.

Another critical, unprecedented trend is the development of amplification-free detection methods for DNA/RNA analysis in FET biosensors. Traditional techniques, such as PCR, involve time-consuming and expensive amplification steps. FET biosensors offer the potential for direct and label-free detection of DNA/RNA molecules without amplification [15]. This trend aims to simplify and streamline the analysis process, reducing assay time and improving accuracy. Researchers are optimizing the probe design, surface functionalization, and signal transduction mechanisms to achieve ultra-sensitive and specific detection of DNA/RNA targets. Amplification-free DNA/RNA detection in FET biosensors holds great promise for various applications, including genetic diagnostics, personalized medicine, and infectious disease detection [16].

Furthermore, integrating non-silicon FET biosensors with complementary metal-oxide-semiconductor (CMOS) technology is an emerging direction that enables on-chip signal processing, multiplexing, and system integration [8]. Silicon-based CMOS technology, which is widely used in microelectronics and is now matured, provides a platform for seamlessly integrating FET biosensors with readout circuitry, analog-to-digital converters, and wireless communication modules; however, novel FETs such as graphene have a long way to go before they reach the same point. This integration enables real-time data acquisition, analysis, and transmission, making novel ultra-sensitive FET biosensors more user-friendly and applicable for point-of-care diagnostics and remote monitoring. CMOS-compatible fabrication processes of these novel BioFETs allow for cost-effective production and scalability, paving the way for large-scale deployment and commercialization.

Novel silicon FET structures relying on the foundry process are receiving more attention as they provide unique opportunities for novel applications. We used an open-gate junction FET (OG-JFET) for the first time for direct DNA sensing in a reference-electrode-free mode [17]. It has enormous potential for DNA data storage applications, enabling the storage and monitoring of a mass amount of DNA with a good retrieval capability. The open-gate sensing area has led to the introduction of a new microfluidic scheme, as well as oral neutrophil detection and pH measurement [18,19,20].

## 3. Comments on the Future Landscape of BioFETs

The future landscape of BioFETs holds immense promise, with several exciting prospects on the horizon. As the field continues to advance, a key area of focus will be the development of multiplexed BioFET devices capable of simultaneously detecting multiple analytes. This will enable comprehensive and efficient analysis of complex biological samples, opening up new possibilities in clinical diagnostics, environmental monitoring, and biomedical research. Integrating nanoscale structures, such as nanowires and nanotubes, into BioFETs shows great potential for enhancing their sensitivity and specificity. This integration allows for detection at even lower analyte concentrations, thereby expanding their applications. By leveraging nanoscale architectures, BioFETs can provide higher resolution and precision in detecting biomolecules, facilitating breakthroughs in disease diagnosis and treatment monitoring. Moreover, the convergence of BioFET technology with other emerging fields, such as nano/microfluidics and machine learning, will drive the development of innovative sensing platforms. Miniaturized and wearable BioFET devices will enable real-time monitoring of health parameters, paving the way for personalized medicine and remote healthcare. These devices will empower individuals to actively participate in managing their health, while also facilitating continuous data collection for medical professionals.

Integrating BioFETs with high-throughput microfluidic systems will revolutionize sample processing and enable high-throughput analysis. This integration will significantly increase the speed and efficiency of biological assays, making BioFETs valuable tools in biomedical research and drug discovery. Additionally, the application of machine learning algorithms and artificial intelligence will aid in data analysis, pattern recognition, and predictive modeling, enhancing the diagnostic capabilities of BioFET devices. These advanced analytical techniques will enable faster and more accurate interpretation of complex biological data, leading to improved disease diagnosis and personalized treatment strategies. However, to unlock the full potential of BioFETs, several challenges must be addressed. Ensuring the long-term stability, reproducibility, and scalability of these devices is crucial for their widespread adoption in various industries. Standardizing fabrication techniques for novel materials such as graphene and MoS2, as well as assay protocols and data analysis methods, will be essential to enable reliable and consistent results across different laboratories. Collaboration among researchers, industry experts, and regulatory bodies will play a vital role in establishing best practices and guidelines. Furthermore, addressing the ethical, legal, and regulatory aspects of using BioFETs in healthcare and their effect on the environment will be imperative to foster public trust and ensure responsible deployment. Safeguarding patient privacy, data security, and compliance with relevant regulations will be essential considerations in the development and implementation of BioFET-based technologies. The future of BioFETs holds tremendous potential for revolutionizing various fields, from healthcare to environmental monitoring and beyond. Advancements in multiplexing capabilities, nanoscale integration, and convergence with other disciplines will propel the development of innovative sensing platforms. By overcoming technical challenges and addressing ethical considerations, BioFETs have the power to transform diagnostics, personalized medicine, and biomedical research, leading to improved outcomes and a better understanding of the complex biological world we inhabit.

## 4. BioFETs as Promising Biosensors for COVID-19 and Future Pandemics

Many studies have reported methods and technologies developed for detecting and treating COVID-19 that can be extended to future outbreaks. The COVID-19 pandemic has widely utilized rapid antigen tests (RATs) to detect the SARS-CoV-2 antigen [21]. These tests have been valuable due to their simplicity and rapid results. Vaccination has also played a crucial role in disease control, with various vaccine types being developed and administered globally and innovative approaches being implemented for assessing other health concerns [22,23,24]. To prepare for future pandemics, it is essential to create affordable, accurate, and adaptable biosensors to enhance screening speed. These biosensors can offer protein-based detection strategies, complement nucleic acid-based tests, and enable rapid and large-scale screening [25,26].

Furthermore, the need for commercialized diagnostic tests that can detect infections at the earliest stages and monitor disease progression remains vital [25,26]. Considering the above requirements, technologies such as FET biosensors hold promise for developing fast, reliable, cost-effective, and portable point-of-care diagnostic tests. These advancements in biosensing technologies will not only aid in combating COVID-19, but will also help detect and manage future infectious diseases [8,25]. In one of our works, we outlined the strategies for developing FET sensors for infectious disease monitoring, explaining all the necessary steps for designing the structure, biological sensing layer, and readout circuit [8]. In our other work, we explained how novel materials like graphene and different novel chemistries are being used with FETs to develop COVID-19 spike protein detection, which has significantly improved the limit of detection and the sensing performance [9].

## 5. Conclusions

BioFETs are undergoing remarkable advancements and finding transformative applications in various fields. Multiplexed BioFET devices; integration with nanoscale structures and 2D materials like graphene and MoS2; convergence with emerging fields such as nanofluidics, machine learning, and artificial intelligence; and addressing challenges in mass production are all shaping the trajectory of this technology. Enhancing the surface chemistries on the FET gate’s dielectric, such as SiO_2_, Si_3_N_4_, and Al_2_O_3_, is crucial for improving repeatability and reducing inherent drift. This is particularly important for reliable detection in complex biofluids like blood, where a high ionic concentration and non-specific bonding pose challenges. By leveraging the power of BioFETs, we can envision highly sensitive, portable, and versatile biosensing platforms that drive advancements in healthcare, diagnostics, and environmental monitoring. We further discussed the importance of BioFETs for developing next-generation biosensors for rapidly detecting infectious diseases like COVID-19. The novel materials being used in BioFETs are promising candidates to revolutionize virus RNA/DNA detection towards developing quantitative on-the-spot screening, which will help immensely in controlling future pandemics. These advancements can enhance individuals’ well-being and contribute to our planet’s sustainability.
